# Machine-Learning Analysis of Voice Samples Recorded through Smartphones: The Combined Effect of Ageing and Gender

**DOI:** 10.3390/s20185022

**Published:** 2020-09-04

**Authors:** Francesco Asci, Giovanni Costantini, Pietro Di Leo, Alessandro Zampogna, Giovanni Ruoppolo, Alfredo Berardelli, Giovanni Saggio, Antonio Suppa

**Affiliations:** 1 Department of Human Neurosciences, Sapienza University of Rome, 00185 Rome, Italy; francesco.asci@uniroma1.it (F.A.); alessandro.zampogna@uniroma1.it (A.Z.); alfredo.berardelli@uniroma1.it (A.B.); 2 Department of Electronic Engineering, University of Rome Tor Vergata, 00133 Rome, Italy; costantini@uniroma2.it (G.C.).; pietro.dileo@alumni.uniroma2.eu (P.D.L.); saggio@uniroma2.it (G.S.); 3 Department of Sense Organs, Otorhinolaryngology Section, Sapienza University of Rome, 00185 Rome, Italy; giovanni.ruoppolo@uniroma1.it; 4 IRCCS Neuromed, 86077 Pozzilli (IS), Italy

**Keywords:** ageing, gender, machine learning, support vector machine, voice analysis

## Abstract

Background: Experimental studies using qualitative or quantitative analysis have demonstrated that the human voice progressively worsens with ageing. These studies, however, have mostly focused on specific voice features without examining their dynamic interaction. To examine the complexity of age-related changes in voice, more advanced techniques based on machine learning have been recently applied to voice recordings but only in a laboratory setting. We here recorded voice samples in a large sample of healthy subjects. To improve the ecological value of our analysis, we collected voice samples directly at home using smartphones. Methods: 138 younger adults (65 males and 73 females, age range: 15–30) and 123 older adults (47 males and 76 females, age range: 40–85) produced a sustained emission of a vowel and a sentence. The recorded voice samples underwent a machine learning analysis through a support vector machine algorithm. Results: The machine learning analysis of voice samples from both speech tasks discriminated between younger and older adults, and between males and females, with high statistical accuracy. Conclusions: By recording voice samples through smartphones in an ecological setting, we demonstrated the combined effect of age and gender on voice. Our machine learning analysis demonstrates the effect of ageing on voice.

## 1. Introduction

Human voice represents a complex biological signal resulting from the dynamic interaction of vocal folds adduction/vibration with pulmonary air emission and flow through resonant structures [1]. Physiologic ageing leads to specific changes in the anatomy and physiology of all structures involved in the production and modulation of the human voice [2,3,4,5,6,7,8,9,10,11,12,13,14]. Hence, a possible approach to evaluate the effect of physiological ageing in humans would include the analysis of voice.

Early seminal studies aimed to characterize age-related changes in voice have used qualitative tools consisting of a perceptual examination of voice recordings [3]. These studies have demonstrated that physiologic ageing induces a variable combination of effects on voice including reduced intensity and phonation time, and a general worsening of voice quality due to hoarseness and vocal fatigue [1,15,16,17]. Some authors have also used more advanced quantitative tools for recording and analyzing voice and thus for achieving an objective examination of age-related changes of voice [1]. Objective voice analysis commonly includes several acoustic parameters calculated in the time-domain such as the jitter, the shimmer, the signal to noise ratio (SNR) and the harmonic to noise ratio (HNR) [18] or spectral analysis measures calculated in the frequency-domain such as the fundamental frequency (fo) [19,20]. More recently, cepstral analysis has been recognized as a methodologic evolution of the spectral analysis resulting from a mathematical transformation from the domain of frequency to quefrency. The cepstral analysis allows for calculating innovative variables such as the cepstral prominence peak smoothed (CPPs) [21,22]. Spectral and cepstral analyses have demonstrated that physiological ageing induces changes in several voice parameters including the fo, the SNR, the HNR, and finally the CPPs [1,20,23]. However, although spectral/cepstral analysis allows measuring age-related changes in specific voice features, it failed to provide a detailed examination of the complex and dynamic interaction of voice features which characterize the physiologic ageing of voice [1,23].

The most recent approach used to assess physiologic ageing in healthy subjects consists of the objective voice analysis based on machine learning algorithms [24,25,26,27,28]. Machine-learning is a novel and robust method commonly applied to classify complex variables obtained from large datasets [29,30,31]. More in detail, machine learning can be applied to predict outcomes from recurring patterns of features within various types of multidimensional data sets [32]. Several authors have applied automatic classifiers based on machine learning analysis on voice recordings to classify healthy subjects according to their age and gender [24,25,26,27,28,33,34,35,36,37,38]. More recently, to further improve the overall accuracy of the machine learning analysis, several studies have included an increasing number of voice features in the datasets [24,25,26,27,28] and compared the performance of different machine learning algorithms [37,38].

In this study, we examined the combined effect of the age- and gender-related factors on voice features through machine learning. Also, previous studies have not compared the performances of the machine learning analysis of voice samples obtained during the sustained emission of a vowel or a sentence, by using the receiver operating characteristic (ROC) curve. So far voice samples have been only collected in a laboratory setting by using dedicated technological instruments consisting of hi-tech audio recorders which require expert supervision [1]. Currently available smartphones and information technology (IT) services have allowed to record and analyze a large number of health parameters in free-living scenarios [39]. The use of a smartphone to record high-quality voice samples would simplify the procedures of recordings, allowing to acquire and analyze a large amount of data. Further advantages of doing recordings using smartphone consist of the building up of a more ecologic scenario compared to the laboratory setting, thus helping to overcome possible voice changes due to supervised conditions.

In this cross-sectional study, we collected voice samples recorded through smartphones in two independent groups of healthy participants with different ages. We used machine learning algorithms to investigate the effect of physiologic ageing on voice. To evaluate the combined effect of age and gender on voice, we also examined the voice samples recorded by females and males from different ages, using machine learning. To verify whether age-related changes of the voice depends on specific speech tasks, we examined and compared the voice recordings during the sustained emission of a vowel and a sentence. All analyses included ROC curves and a detailed description of the statistical output including accuracy, sensibility, specificity, and area under the curve (AUC).

## 2. Materials and Methods

### 2.1. Subjects

We recruited an overall group of 261 healthy subjects (HS) (112 males and 149 females; mean age ± SD 41.0 ± 18.7 years, range 15–85). Subjects were then divided into two independent sex-matched groups according to age: younger adults (YA) (number 138; 65 males and 73 females; mean age ± SD 25.1 ± 3.1 years, range 15–30), and older adults (OA) (number 123; 47 males and 76 females; mean age ± SD 58.9 ± 11.0 years, range 40–85). All the participants were recruited at the Department of Human Neurosciences, Sapienza University of Rome, Italy. All subjects were non-smokers, native Italian-speakers. Participants did not manifest cognitive or mood impairment nor bilateral/unilateral hearing loss, respiratory disorders, and other disorders affecting the vocal cords. Also, participants did not manifest gastro-esophageal reflux disease, acute or chronic gastritis, or other gastrointestinal disorders possibly affecting the emission of the voice. At the time of the study, all the YA completed the pubertal development. Participants took no drugs acting over the central nervous system at the time of the study. Participant demographic features are summarized in Table 1 and reported in detail in Supplementary Materials Tables S1 and S2. Participants gave consent to the study, which was approved by the institutional review board following the Declaration of Helsinki.

### 2.2. Voice Recordings

The recording session started by asking participants to sit on a chair in the middle of a silent room. Subjects were instructed to handle and face a smartphone at about 30 cm from the mouth and then to speak with their usual voice intensity, pitch, and quality. Smartphones currently available in the market (various brands including Apple^®^, Samsung^®^, Huawei^®^, Xiaomi^®^ and Asus^®^) were used for voice recordings. The recording session consisted of two separate speech-tasks, the former including the sustained emission of a vowel and the latter consisting of a sample of connected-speech. More in detail, patients were first asked to produce the sustained emission of the vowel/e/for 5 s and then to read the following Italian phonetically balanced sentence: “Nella casa in riva al mare maria vide tre cani bianchi e neri.” To simplify the procedures of home-made audio recording, all participants were asked to save the audio tracks in mp4 format at the end of the recording session. Participants were then asked to send voice samples by e-mail to our institutional mail server, which was protected and accessible only by the authors. Lastly, voice recordings were separated in audio tracks containing each of the two speech-tasks, through a segmentation procedure included in dedicated software for audio-editing (Audacity^®^) [40].

### 2.3. Machine-Learning Analysis

The machine-learning analysis consisted of specific and standardized algorithms of artificial intelligence [41,42,43,44]. We converted all the audio tracks from mp4 into Wav format (sampling frequency: 44.1 kHz; bit depth: 16 bit), before submitting data to OpenSMILE, a dedicated software for the pre-process of feature extraction (OpenSMILE; audEERING GmbH, Munich, Germany) [45]. For each voice sample, 6139 voice features were extracted by using a modified INTERSPEECH2016 Computational Paralinguistics Challenge (IS ComParE 2016) feature dataset [44]. IS ComParE 2016 contains voice features calculated using computational functionals (e.g., mean, quartiles, percentiles, position of max/min, linear regression) over acoustic low-level descriptors (LLDs), including those related to the energy, spectrum, cepstrum of the signal [44,46], and also including the Mel-Frequency Cepstral Coefficients [47,48], RASTA-PLP Coefficients [49], jitter, shimmer, sound quality descriptors, and prosodic features. Given that the IS ComParE 2016 features dataset does not contain the CPPs, the HNR, and SNR, we additionally extracted these features through specific home-made algorithms (MATLAB, The Math Works, Inc., Version R2020a, Natick, MA, USA, 2020) [21,50,51]. Then, the CPPs, HNR, and SNR were added to the IS ComParE 2016 feature dataset using Wolfram Mathematica (Wolfram Research, Inc., Mathematica, Version 12.1, Champaign, IL, USA, 2020).

To identify a small subset of relevant features for the objective analysis of voice ageing [52], the extracted voice features underwent feature selection using the correlation features selection (CFS) algorithm [53]. Through CFS, we selected voice features highly correlated with the class, thus removing the irrelevant and redundant features from the original dataset. Selected features were ranked by using the correlation attribute evaluation (CAE) algorithm, which evaluates and ranks all the attributes in order of relevance, according to Pearson’s correlation method. To further increase the accuracy of results, we applied the Fayyad & Irani’s discretization method to the features’ values [54]. Discretization is an optimization procedure consisting in modifying the values and the distribution of the features, by calculating the best splitting point from the two classes and assigning a binary value to the features, in two groups.

**Figure 1:**
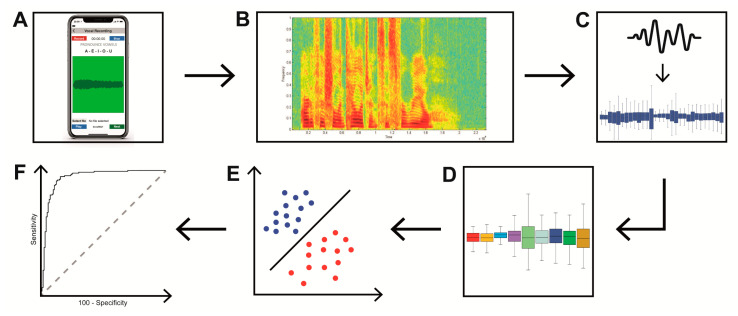
Experimental procedures. (**A**) Smartphone recording of voice samples of sustained emission of vowel and a sentence. (**B**) Acoustic voice spectrogram. (**C**) Procedures of features extraction, (**D**) features selection, and (**E**) classification obtained through the SVM. (**F**) Receiver operating characteristic (ROC) analysis used to perform the statistics.

After pre-processing procedures, we started the machine learning analysis by using the support vector machine (SVM) classifier. To train the SVM, we considered only the first twenty most relevant features ranked by the CAE. This approach was applied to reduce the number of selected features needed to perform the machine learning analysis. Specifically, the SVM was trained using the sequential minimal optimization (SMO) method, which is considered a fast and efficient machine learning algorithm to implement an SVM classifier [55]. All the classifications were made using a 5-or 10-folds cross-validation, depending on the number of the instances (voice samples) contained in the examined dataset. Both the feature selection and the classification were performed by dedicated software that contains a collection of algorithms for data analysis and predictive modelling (Weka, Waikato Environment for Knowledge Analysis, University of Waikato, New Zealand) [53,56]. The experimental procedures are summarized in Figure 1.

### 2.4. Statistical Analysis

The normality of the demographic and anthropometric variables in YA and OA was assessed using the Kolmogorov-Smirnov test. Mann-Whitney U test was used to compare demographic scores in YA and OA. ROC analyses were performed to identify the optimal diagnostic cut-off values of SMO (selected features), calculated during the sustained emission of the vowel as well as during the emission of the sentence, for discriminating between (1) YA and OA; (2) female YA and OA; (3) male YA and OA; (4) male and female YA and finally; (5) male and female OA. Cut-off values were calculated as the point of the curves with the highest Youden index (sensitivity + specificity − 1) to maximize the sensitivity and specificity of the diagnostic tests. The positive and negative predictive values were also calculated. According to standardized procedures [57], we compared the area under the curves (AUCs) in the ROC curves calculated from SMO (selected features) to verify the optimal test for discriminating within the subgroups. All ROC analyses were performed using WEKA and Wolfram Mathematica. *p* < 0.05 was considered statistically significant. Unless otherwise stated, all values are presented as mean ± standard deviation (SD). Statistical analyses were performed using Statistica version 10 (StatSoft, Inc) and Wolfram Mathematica.

### 2.5. Data Availability

The anonymized database used in the current study is available from the corresponding author on reasonable request for a limited time-window of 3 months after publication.

## 3. Results

The Kolmogorov-Smirnov test showed that demographic and anthropometric parameters were normally distributed in the YA and OA as well as in female and male YA and OA subjects (*p* > 0.05 for all analyses). Mann-Whitney U test showed increased weight and BMI and decreased height values in OA subjects compared with YA (*p* < 0.05 for all comparisons)(Table 1, Supplementary Materials Tables S1 and S2).

### 3.1. YA and OA

**Figure 2:**
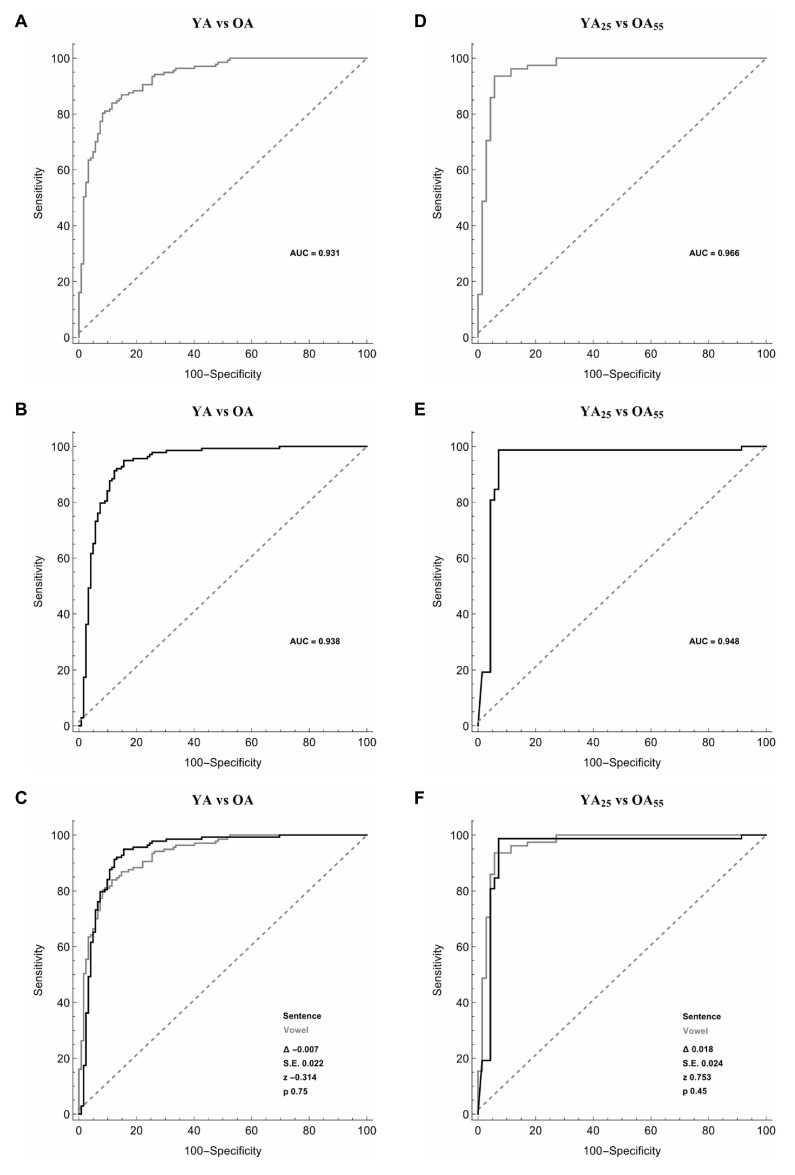
Receiver operating characteristic (ROC) curves used to differentiate younger adults (YA) vs. older adults (OA) (left column, panels (**A**–**C**)) and younger adults ≤ 25 years (YA_25_) vs. older adults ≥ 55 years (OA_55_) (right column, panels (**D**–**F**)) during the sustained emission of a vowel (grey line) (panels (**A**,**D**)), the sentence (black line) (panels (**B**,**E**)) and the comparison between the vowel and the sentence (panels (**C**,**F**)).

When discriminating YA and OA, the artificial classifier based on SMO using selected features allowed us to achieve a significant diagnostic performance of our test. When comparing the 20 most relevant selected features extracted from the sustained emission of the vowel, ROC curve analyses identified an optimal diagnostic threshold value of 0.50 (associated criterion), when applying discretization and 10-folds cross-validation (Y.I = 0.72). Using this cut-off value, the performance of our diagnostic test was: sensitivity = 86.9%, specificity = 85.2%, PPV = 86.9%, NPV = 85.2%, accuracy = 86.1%, and AUC = 0.931 (Figure 2A, Table 2). Furthermore, when comparing 20 selected features extracted from the sustained emission of the sentence, ROC curve analyses identified an optimal diagnostic threshold value of 0.50, when applying discretization and 10-folds cross-validation (Y.I = 0.77). Using this cut-off value, the performance of our diagnostic test was: sensitivity = 89.1%, specificity = 87.7%, PPV = 89.1%, NPV = 87.7%, accuracy = 88.5%, and AUC = 0.938 (Figure 2B, Table 2). The two ROC curves obtained during the emission of the vowel and the sentence were comparable (the difference between AUCs = −0.007, z = −0.314, SE = 0.022, *p* = 0.75) (Figure 2C).

To reduce excessive age dispersion, and thus perform a more consistent analysis of voice ageing, in a further analysis we compared the voice recordings collected from two subgroups of YA and OA. Moreover, in detail, among YA, we considered a subgroup of 79 YA with age ≤ 25 years (YA_25_) (31 males and 41 females; mean age ± SD 22.9 ± 2.2 years, range 15–25), whereas, among OA, we selected a subgroup of 71 OA with age ≥ 55 years (OA_55_) (21 males and 50 females; mean age ± SD 66.4 ± 8.1 years, range 55–85). When comparing the sustained emission of the vowel and the sentence in YA_25_ and OA_55_ we achieved further improvement in the results as shown by the ROC curve analysis. More in detail, when comparing 20 selected features extracted from the sustained emission of the vowel, ROC curve analyses identified optimal diagnostic threshold value of 0.59, when applying discretization and five-folds cross-validation (Y.I = 0.86). Using this cut-off value, the performance of our diagnostic was: sensitivity = 93.6%, specificity = 92.9%, PPV = 93.6%, NPV = 92.9%, accuracy = 93.2%, and AUC = 0.966 (Figure 2D, Table 2). Also, when comparing 20 selected features extracted from the sustained emission of the sentence, ROC curve analyses identified an optimal diagnostic threshold value of 0.52, when applying discretization and five-folds cross-validation (Y.I = 0.91). Using this cut-off value, the performance of our diagnostic test was: sensitivity = 92.8%, specificity = 98.5%, PPV = 98.7%, NPV = 91.4%, accuracy = 95.3%, and AUC = 0.984 (Figure 2E, Table 2). Again, the two ROC curves obtained during the emission of the vowel and the sentence were comparable (the difference between AUCs = 0.018, z = 0.753, SE = 0.024, *p* = 0.45) (Figure 2F).

### 3.2. Female YA and Female OA

**Figure 3:**
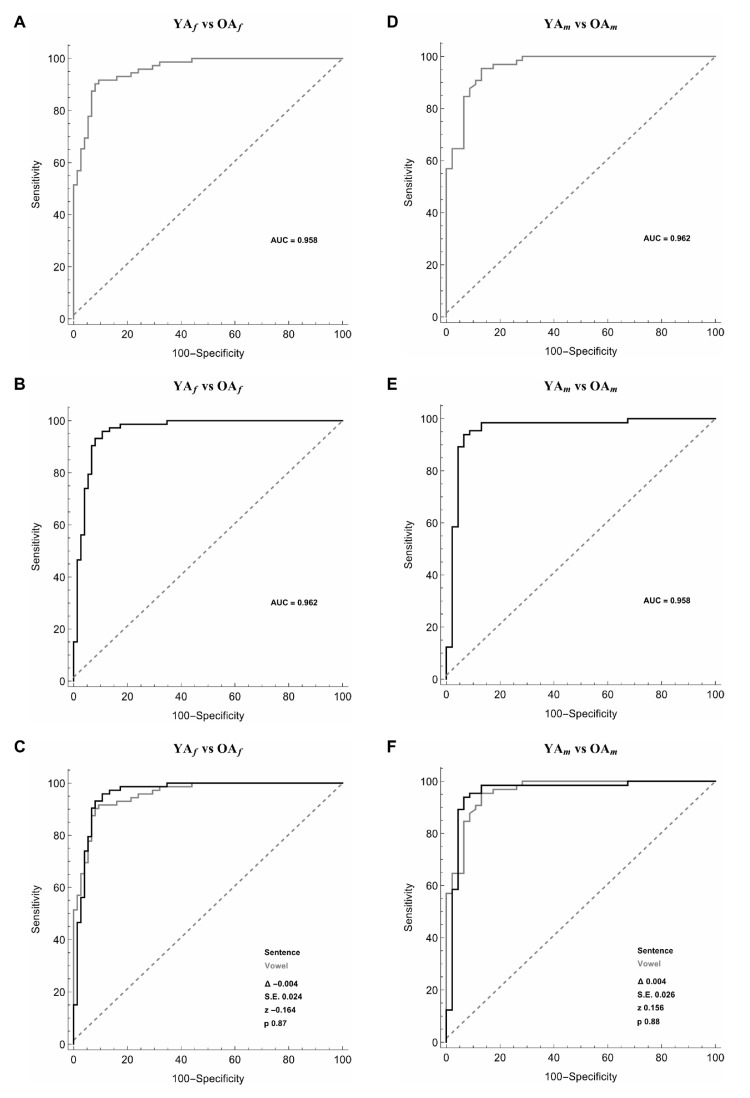
Receiver operating characteristic (ROC) curves used to differentiate female younger adults (YA*_f_*) and female older adults (OA*_f_*) (left column, panels (**A**–**C**)) and male younger adults (YA*_m_*) and male older adults (OA*_m_*) (right column, panels (**D**–**F**)) during the sustained emission of a vowel (grey line) (panels (**A**,**D**)), the sentence (black line) (panels (**B**,**E**)), and the comparison between the vowel and the sentence (panels (**C**,**F**)).

In the comparison of female YA and OA, the artificial classifier based on SMO achieved a significant diagnostic performance. More in detail, when comparing 20 selected features extracted from the sustained emission of the vowel, ROC curve analyses identified an optimal diagnostic threshold value of 0.57, when applying discretization and five-folds cross-validation (Y.I = 0.81). Using this cut-off value, the performance of our diagnostic test was: sensitivity = 90.3%, specificity = 90.7%, PPV = 90.3%, NPV = 90.7%, accuracy = 90.5% and AUC = 0.958 (Figure 3A, Table 2). Also, when examining the sustained emission of the sentence, ROC curve analyses identified optimal diagnostic threshold value of 0.66, when applying discretization and five-folds cross-validation (Y.I = 0.85). Using this cut-off value, the performance of our diagnostic test was: sensitivity = 91.9%, specificity = 93.2%, PPV = 93.2%, NPV = 92.0%, accuracy = 92.6%, and AUC = 0.962 (Figure 3B, Table 2). The two ROC curves obtained during the emission of the vowel and the sentence were similar (the difference between AUCs = −0.004, z = −0.164, SE = 0.024, *p* = 0.87) (Figure 3C).

### 3.3. Male YA and Male OA

In the comparison of male YA and OA, the artificial classifier based on SMO using 20 selected features achieved a significant diagnostic performance. When comparing selected features extracted from the sustained emission of the vowel, ROC curve analyses identified optimal diagnostic threshold value of 0.53, when applying discretization and five-folds cross-validation (Y.I = 0.82). Using this cut-off value, the performance of our diagnostic test was: sensitivity = 91.0%, specificity = 90.9%, PPV = 93.8%, NPV = 87.0%, accuracy = 91.0% and AUC = 0.962 (Figure 3D, Table 2). Also, when examining the sustained emission of the sentence, ROC curve analyses identified an optimal diagnostic threshold value of 0.52, when applying discretization and five-folds cross-validation (Y.I = 0.87). Using this cut-off value, the performance of our diagnostic test was: sensitivity = 91.3%, specificity = 95.2%, PPV = 96.9%, NPV = 87.0%, accuracy = 92.8%, and AUC = 0.958 (Figure 3E, Table 2). The difference between the two ROC curves obtained during the emission of the vowel and the sentence was not significant (the difference between AUCs = 0.004, z = 0.156, SE = 0.026, *p* = 0.88) (Figure 3F).

### 3.4. Male and Female YA

**Figure 4:**
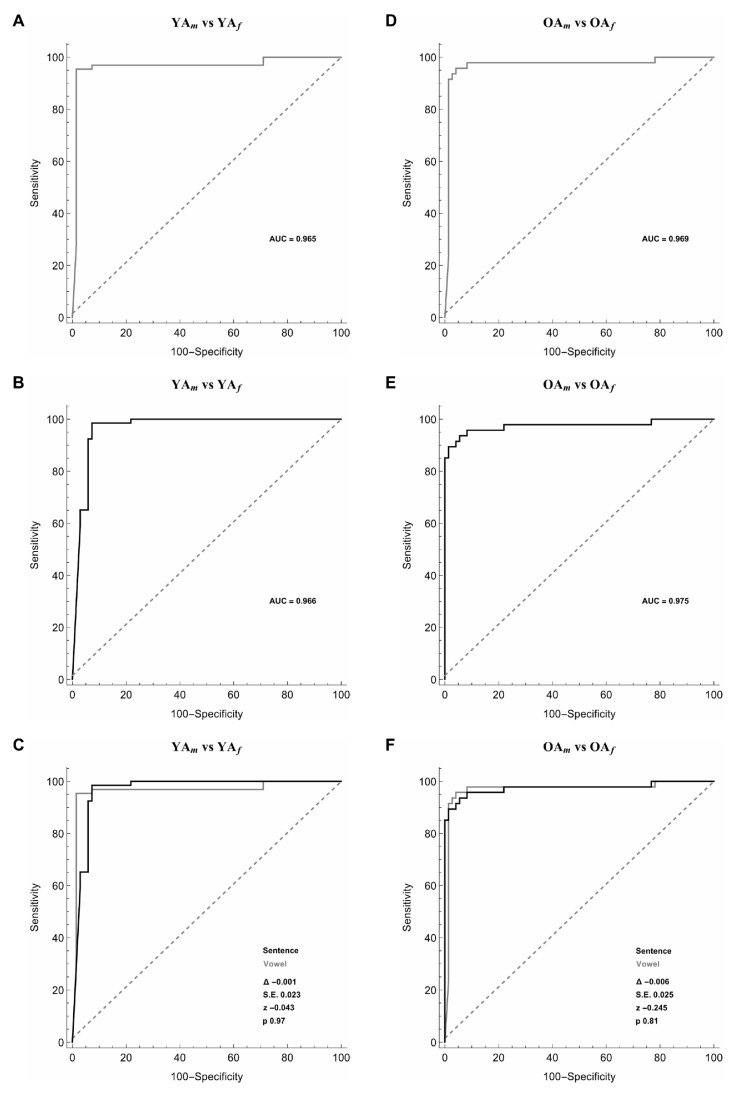
Receiver operating characteristic (ROC) curves used to differentiate female Younger Adults (YA*_f_*) and male Younger Adults (YA*_m_*) (left column, panels (**A**–**C**)) and female older adults (OA*_f_*) and male older adults (OA*_m_*) (right column, panels (**D**–**F**)) during the sustained emission of a vowel (grey line) (panels (**A**,**D**)), the sentence (black line) (panels (**B**,**E**)) and the comparison between the vowel and the sentence (panels (**C**,**F**)).

In the analysis of male vs. female YA, the artificial classifier based on SMO achieved a significant diagnostic performance. More in detail, when comparing 20 selected features extracted from the sustained emission of the vowel, ROC curve analyses identified an optimal diagnostic threshold value of 0.69, when applying discretization and 5-folds cross-validation (Y.I = 0.91). Using this cut-off value, the performance of our diagnostic test was: Sensitivity = 95.4%, Specificity = 95.7%, PPV = 95.4%, NPV = 95.7%, Accuracy = 95.5% and AUC = 0.965 (Figure 4A, Table 2). Also, when analyzing the sustained emission of the sentence, ROC curve analyses identified an optimal diagnostic threshold value of 0.61, when applying discretization and 5-folds cross-validation (Y.I = 0.89). Using this cut-off value, the performance of our diagnostic test was: sensitivity = 90.3%, specificity = 98.4%, PPV = 98.5%, NPV = 89.9%, accuracy = 94.1%, and AUC = 0.966 (Figure 4B, Table 2). The two ROC curves obtained during the emission of the vowel and the sentence were comparable (the difference between AUCs = −0.001, z = −0.043, SE = 0.023, *p* = 0.97) (Figure 4C).

### 3.5. Male and Female OA

When differentiating male and female OA, the artificial classifier based on SMO achieved a significant diagnostic performance. More in detail, when comparing 20 selected features extracted from the sustained emission of the vowel, ROC curve analyses identified an optimal diagnostic threshold value of 0.74, when applying discretization and five-folds cross-validation (Y.I = 0.87). Using this cut-off value, the performance of our diagnostic test was: sensitivity = 89.4%, specificity = 97.1%, PPV = 95.5%, NPV = 93.2%, accuracy = 94.2%, and AUC = 0.969 (Figure 4D, Table 2). Also, when examining the sustained emission of the sentence, ROC curve analyses identified an optimal diagnostic threshold value of 0.63, when applying discretization and five-folds cross-validation (Y.I = 0.86). Using this cut-off value, the performance of our diagnostic test was: sensitivity = 89.8%, specificity = 95.8%, PPV = 93.6%, NPV = 93.2%, accuracy = 93.3%, and AUC = 0.975 (Figure 4E, Table 2). The two ROC curves obtained during the emission of the vowel and the sentence were comparable (the difference between AUCs = −0.006, z = −0.245, SE = 0.025, *p* = 0.81) (Figure 4F).

## 4. Discussion

In this study, we found that machine learning analysis of voice samples recorded through smartphones correctly discriminates between YA and OA. We have also demonstrated that our voice analysis accurately discriminates females and males in both groups. By comparing male and female YA, as well as male and female OA, we have also examined in detail the combined effect of age and gender on voice. Accordingly, by using machine learning analysis, in this study we have demonstrated the effect of ageing and gender on voice.

To collect homogeneous and high-quality recordings, we have carefully controlled for several methodological factors. All participants were native Italian speakers. To exclude confounding related to the acute and chronic effects of smoking on the physiology of the vocal folds, lungs, and resonant structures, we have included in the study only non-smokers. By contrast, we excluded subjects with cognitive or mood impairment or those taking drugs acting on the central nervous system at the time of the study. We also excluded from the study cohort subjects with bilateral/unilateral hearing loss, respiratory disorders, and other pathological conditions directly or indirectly affecting the vocal cords. The age range considered for the YA group was based on the definition of young subjects provided by the World Health Organization [58]. Accordingly, all the YA participants completed the pubertal development. By contrast, the age range considered for the OA group was set to include subjects in the middle and late adulthood [59]. In this study, we excluded voice recordings from subjects in the early adulthood (30–40 years) in order to better separate the study cohort into two independent subgroups of different ages. Lastly, all voice samples were collected through smartphones able to save audio tracks in mp4 format.

The main novelty of the study consists of the acquisition and analysis of voice samples collected through smartphones. Indeed, although a few studies have previously used smartphones to collect voice samples in patients with voice disorders [60,61,62], so far no authors have used this methodological approach to examine age-related changes of voice. The use of smartphones allows a simplified procedure of voice recordings and open to the acquisition of a large amount of data collected in an ecologic scenario.

### 4.1. The Effect of Ageing on Voice

The first finding of our study is that the objective voice analysis based on machine learning can distinguish YA and OA subjects, with a high level of accuracy as demonstrated by our ROC curve analyses. The accuracy of the algorithm tended to improve further when comparing the YA and OA subjects with a narrower age-band (YA_25_ and OA_55_). Furthermore, to investigate age-related changes in the human voice in more detail, we have also compared gender-matched groups of YA and OA subjects. Indeed, by comparing females included in the YA and OA groups as well as males included in the YA and OA groups, in separate analyses, we have examined the pure effect of ageing on voice. Our findings fully agree with previous reports demonstrating the effect of ageing on the human voice [24,25,26,27,28,33,34,35,36,37,38]. Early studies based on the qualitative/perceptual evaluation of voice recordings have demonstrated that physiologic ageing leads to several changes in specific characteristics of the human voice [1]. Indeed, as a result of physiologic ageing, voices progressively manifest increased breathiness and hoarseness, reduced speech intensity as well as maximum phonation time [2,3,4,15]. Experimental studies using spectral analysis have confirmed age-related changes in voice by providing new objective measures in the time-domain as well as in the frequency-domain. For instance, both the jitter and the shimmer were higher in OA than in YA subjects [1], the former reflecting the degree of voice hoarseness [63], whereas the latter relates to the degree of the breathiness of the voice [1]. Also, the N/H ratio, which reflects the level of noise of an acoustic signal, also increases in the elderly [18]. Finally, concerning measures in the frequency domain, previous studies using spectral analysis have also shown age-related changes in voice even though with some inconsistency. For instance, in the elderly, the fundamental frequency (f0) decreased [64,65,66,67], increased [68,69,70], or even remain unchanged [71,72,73].

In our study, by applying the ROC curve analysis, we demonstrated in detail the high accuracy of our machine learning analysis in demonstrating age-related changes in the human voice. Our results fit in well with previous studies applying automatic classifiers based on machine learning analysis [24,25,26,27,28,33,34,35,36,37,38]. More in detail, our machine learning algorithm has achieved higher results than those obtained on the INTERSPEECH 2010 age and gender sub-challenge feature set [33,34]. Among machine learning algorithms, the standard and hybrid versions of the SVM (e.g., SVM-GMM) are thought to be both consistent and accurate [33,34,35,38,73]. In our study, SVM achieved relatively high performance with an accuracy of 95.3% in age recognition and of 95.5% in gender recognition, showing comparable or even better results than those obtained in previous reports [33,34,35,38,73]. When comparing our methodological approach to those previously used, it is important to consider that we started with a large dataset of features (more than 6000), adopting dedicated ranking and feature selection algorithms [33,34,35,36,37,38,73]. The advantages of applying those algorithms consist of obtaining smaller dataset of features (only 20 features in our study), easier math handled and with shorter computation time. Moreover, all the previous studies considered only MFCC-, f0-, pitch-, energy-, jitter-, and shimmer-related features [24,25,26,27,28,33,34,35,36,37], with only a study considering non-traditional features including RASTA-PLP coefficients [38]. In addition to the traditional frequency-, jitter-, shimmer-, energy-, spectral, and cepstral-related features, we have also included MFCC and RASTA-PLP coefficients and three additional representative features (HNR, SNR, and CPPs). The inclusion of HNR, SNR, CPPs, and RASTA-PLP coefficients to the general dataset of LLDs allowed us to achieve a more robust analysis. Indeed, these features were frequently included in the 20 most relevant selected features in all the comparisons made by our machine learning algorithm. Also, SNR, CPPs, MFCC-, RASTA coefficients-, fo-, spectral-, and energy-related features specifically changed in the human voice according to physiologic ageing (see Table S3 in supplementary material for a detailed list of the first 20 selected features during the comparison between YA and OA). In our case, particularly the RASTA filtering technique has allowed reducing the irrelevant information introduced into the signal by the microphones or by the background noise [49]. Since in our study each vocal sample was recorded with a different smartphone the use of RASTA filtering made possible to eliminate the effect due to the use of different microphones.

Several age-related changes in physiological functions may explain our findings. The physiological basis underlying our results and those previously obtained with the perceptual and standard objective analysis are prominently related to age-related changes of the phonatory apparatus. These changes are secondary to: Loss of elasticity and tone of the vocal folds and the pharyngeal walls; increase of fat distribution in the neck and the parapharyngeal space; progressive reduction of the secretion of the salivary and mucous glands; thinning of the tongue and loss of teeth with relevant changes in shape and diameter of the oral cavity [5]. Moreover, at a cellular and molecular level, physiological ageing leads to thinning of the laryngeal epithelium, loss of the elastic chord component, and increase in the collagen fibers/elastic fibers ratio which in turn decrease vocal folds viscoelasticity [6,7,8,9,10,11,12,13,14]. Also, the myelin fiber density of the superior and recurrent laryngeal nerve progressively reduces with age leading to an alteration of the intrinsic reflex tone and muscle flaccidity [74,75]. Besides age-related changes in specific components of the phonatory apparatus, voice can be influenced also by additional anthropometric factors including weight and height of the subjects. In this study, we found that OA subjects had increased weight and BMI and decreased height values compared with YA. Although our methodological approach does not allow to clarify the link between any of the voice features selected by the SMO and age-related changes in specific components of the phonatory apparatus or anthropometric factors, we believe that our machine learning analysis of the human voice provides objective evaluation of the human ageing.

### 4.2. The Effect of Gender on Voice

Our machine learning analysis allowed us also to examine in detail the effect of gender on voice. Our machine learning analysis differentiated female and male YA as well as female and male OA with high accuracy. It is known that gender leads to additional sources of variability in voice features. Previous perceptual and objective studies of the human voice have shown that before the pubertal age, males and females have a rather similar vocal pitch. During puberty, the male voice typically deepens an octave, while the female voice usually deepens only by a few tones. Thus, before puberty, the voice examination does not show any difference between males and females, whereas, in the adulthood, the examiner can usually recognize the gender of the speaker [18,63,64,65,67,68,71,72,73]. The physiologic basis of differences in voice parameters between males and females relies on several physiologic and anatomic issues. The hormones grow the larynx and the vocal folds in both males and females, but in males, the growth is rather prominent. Then, in women during the menopausal phase, the level of estrogen hormone decreases along with an increase in androgens. As a result, the thickness of the vocal cords increases and leads to a deeper tone of voice. A complementary phenomenon occurs in males during andropause, characterized by a drop in the level of androgens and a relative increase of the estrogen/androgen ratio [5,76]. Our findings agree with previous findings from perceptive and quantitative voice studies further demonstrating that voice objectively differs in females and males [1]. However, our machine learning analysis does not provide evidence for a strict relation between any of the voice features here considered and specific gender-related changes in the phonatory apparatus.

Another important finding of our study concerns the comparable results achieved when examining voice samples collected during the emission of the vowel and the sentence [24,77]. This finding suggests the comparable ability of machine learning to recognize voice changes due to the combined effect of ageing and gender, during the sustained emission of a vowel as well as a sentence. We suggest, however, that compared to the recording of a sentence, voice samples including the sustained emission of a vowel would be more practical and more reliable thus improving voice analyses among the different languages.

A final comment concerns how relevant is the objective evaluation of ageing processes in humans [78]. Age can be classified into “chronological” and “biological” components [79], the former referring to the actual amount of years of a subject, whereas the latter reflects the amount of age-related changes in various physiological functions in the same subject. The physiologic ageing represents a gradual and continuous process reflecting the interaction between genetic and environmental factors, and leading to the progressive decline of physical, psychological, and social functions [80]. To date, no standardized biomarkers of physiologic ageing are currently available. We, therefore, believe that our voice analysis with machine learning would provide a novel and advanced tool possibly helpful for quantifying the individual “biological” age of a single subject [81,82]. The objective voice analysis would also allow to better discriminate and monitor processes of physiological as well as pathological ageing.

A possible limitation of this study is the reduced sample of voice recordings undergoing machine learning analysis. However, the level of significance of our results in all the comparison is relatively high. We did not record voices in young females under different phases of the menstrual cycle thus not excluding the possible effect of hormones on voices. The intrinsic variability in the brand and model of the smartphones used to record voice samples (e.g., variability related to microphones and recording algorithms) would have affected our results. For instance, depending on the specific smartphone used, mp4 audio files can be compressed through different audio coding standards for lossy or lossless digital audio compression (e.g., AAC—advanced audio coding; Apple Lossless Audio Codec—ALEC, or Free Lossless Audio Codec—FLAC). Hence, we cannot exclude that the heterogeneity in the brand and model of the smartphones also increased the variability of our data. Also, since in the present study we did not record voice samples serially, we cannot exclude variability in voice recordings due to daily fluctuations in voice parameters. Furthermore, our study did not include the longitudinal evaluation of voice recordings in the same subjects. This study design although theoretically feasible is technically difficult. Hence, in the present study, the lack of a follow-up evaluation of voice recordings did not allow us to clarify intra-subject age-related changes in the human voice. Lastly, we cannot fully exclude that the increased weight and BMI, and the decreased height observed in OA subjects would have contributed at least in part to our findings [83].

## 5. Conclusions

Advanced voice analysis based on machine-learning performed on voice samples collected using smartphones can distinguish between younger and older healthy subjects, thus objectively evaluating the effect of physiologic ageing on the voice in humans. Our voice analysis is also able to discriminate between females and males from YA and OA groups, thus demonstrating the interaction between ageing- and gender-related factors in determining the human voice. Future cohort studies comparing voice recordings in a larger number of samples of different ages (e.g., large samples of subjects in early, middle and late adulthood) will better examine whether age-related changes in voice can be considered biomarkers of human ageing. Furthermore, we believe that our study would provide new helpful information to clinicians to better distinguish physiologic ageing from pathological changes of the human voice in subjects affected by various speech disorders [77,84].

## Figures and Tables

**Table 1:** Demographic and clinical characteristics of the participants.

Group	Age (years)	Weight (Kg)	Height (cm)	BMI
YA	25.1 ± 3.1	64.5 ± 12.4	171.4 ± 8.5	21.8 ± 3.1
OA	58.9 ± 11.0	66.9 ± 11.9	166.5 ± 9.8	25.2 ± 4.1
YA_25_	22.9 ± 2.2	61.4 ± 10.3	171.0 ± 8.1	20.9 ± 2.5
OA_55_	66.4 ± 8.1	68.6 ± 11.9	163.0 ± 9.1	25.8 ± 4.3
YA*_f_*	24.7 ± 3.0	56.5 ± 7.6	166.2 ± 5.7	20.5 ± 2.7
YA*_m_*	25.5 ± 3.2	73.4 ± 10.7	177.2 ± 7.1	23.3 ± 2.8
OA*_f_*	59.8 ± 10.5	65.7 ± 11.3	161.2 ± 7.3	25.4 ± 4.7
OA*_m_*	58.1 ± 11.3	76.4 ± 9.6	175.0 ± 6.9	25.0 ± 3.1

OA: older adult; OA*_f_*: female OA; OA*_m_*: male OA; YA*_f_*: female YA; YA*_m_*: male YA; YO: younger adult; YO_25_: younger adult ≤ 25 years; OA_55_: older adult ≥ 55 years. Values are expressed as average ± standard deviation.

**Table 2:** Performance of the machine-learning algorithm in all the comparisons.

Comparisons	Speech-Task	Number of Instances	Cross-Validation	Assoc. Criterion	Youden Index	Se (%)	Sp (%)	PPV (%)	NPV (%)	Acc (%)	AUC
YA vs. OA	Vowel	259	10 folds	0.50	0.72	86.9	85.2	86.9	85.2	86.1	0.961
	Sentence	260	10 folds	0.50	0.77	89.1	87.7	89.1	87.7	88.5	0.938
YA_25_ vs. OA_55_	Vowel	148	5 folds	0.59	0.86	93.6	92.9	93.6	92.9	93.2	0.966
	Sentence	148	5 folds	0.52	0.91	92.8	98.5	98.7	91.4	95.3	0.984
YA*_f_* vs. OA*_f_*	Vowel	147	5 folds	0.57	0.81	90.3	90.7	90.3	90.7	90.5	0.958
	Sentence	148	5 folds	0.66	0.85	91.9	93.2	93.2	92.0	92.6	0.962
YA*_m_* vs. OA*_m_*	Vowel	111	5 folds	0.53	0.82	91.0	90.9	93.8	87.0	91.0	0.962
	Sentence	111	5 folds	0.52	0.87	91.3	95.2	96.9	87.0	92.8	0.958
YA*_m_* vs. YA*_f_*	Vowel	134	5 folds	0.69	0.91	95.4	95.7	95.4	95.7	95.5	0.965
	Sentence	135	5 folds	0.61	0.89	90.3	98.4	98.5	89.9	94.1	0.966
OA*_m_* vs. OA*_f_*	Vowel	120	5 folds	0.74	0.87	89.4	97.1	95.5	93.2	94.2	0.969
	Sentence	120	5 folds	0.63	0.86	89.8	95.8	93.6	93.2	93.3	0.975

Acc: accuracy; AUC: area under the curve; NPV: negative predictive value; OA: older adult; OA_55_: older adult ≥ 55 years; OA*_f_*: female OA; OA*_m_*: male OA; PPV: positive predictive value; Se: sensitivity; Sp: specificity; YA*_f_*: female YA; YA*_m_*: male YA; YO: younger adult; YO_25_: younger adult ≤ 25 years. Instances refer to the number of subjects considered in each comparison. Cross-validation refers to standardized procedures of a machine learning algorithm (see the text for details).

## Supplementary Materials

The following are available online at https://www.mdpi.com/1424-8220/20/18/5022/s1, Table S1: Demographic and anthropometric characteristics of younger adults. Table S2: Demographic and anthropometric characteristics of older adults. Table S3. Ranking of the first 20 features (functionals applied to low-level descriptors) extracted using OpenSMILE and selected using CAE for the comparison between YA and OA, during the emission of the vowel and the sentence. Each feature is identified by four items: (1) family of low-level descriptor (LLD), (2) LLD, (3) functional used to calculate that specific feature and, (4) the value of relevance calculated through CAE algorithm.

## References

[1] Goy H., Fernandes D.N., Pichora-Fuller M.K., van Lieshout P. (2013). Normative Voice Data for Younger and Older Adults. J. Voice.

[2] Kendall K. (2007). Presbyphonia: A review. Curr. Opin. Otolaryngol. Head Neck Surg..

[3] De Araújo Pernambuco L., Espelt A., Balata P.M.M., de Lima K.C. (2015). Prevalence of voice disorders in the elderly: A systematic review of population-based studies. Eur. Arch. Otorhinolaryngol..

[4] Mezzedimi C., Di Francesco M., Livi W., Spinosi M.C., De Felice C. (2017). Objective Evaluation of Presbyphonia: Spectroacoustic Study on 142 Patients with Praat. J. Voice.

[5] Bruzzi C., Salsi D., Minghetti D., Negri M., Casolino D., Sessa M. (2017). Presbiphonya. Acta Biomed..

[6] Gonçalves T.M., Dos Santos D.C., Pessin A.B.B., Martins R.H.G. (2016). Scanning Electron Microscopy of the Presbylarynx. Otolaryngol. Head Neck Surg..

[7] Hirano S., Minamiguchi S., Yamashita M., Ohno T., Kanemaru S.-I., Kitamura M. (2009). Histologic characterization of human scarred vocal folds. J. Voice.

[8] Sato K., Hirano M. (1995). Histologic investigation of the macula flava of the human newborn vocal fold. Ann. Otol. Rhinol. Laryngol..

[9] Chan R.W., Gray S.D., Titze I.R. (2001). The importance of hyaluronic acid in vocal fold biomechanics. Otolaryngol. Head Neck Surg..

[10] Chen X., Thibeault S.L. (2008). Characteristics of age-related changes in cultured human vocal fold fibroblasts. Laryngoscope.

[11] Allah R., Dkhil M., Farhoud E. (2009). Fibroblasts in the human vocal fold mucosa: An ultrastructural study of different age groups. Singap. Med. J..

[12] Hammond T.H., Gray S.D., Butler J., Zhou R., Hammond E. (1998). Age- and gender-related elastin distribution changes in human vocal folds. Otolaryngol. Head Neck Surg..

[13] McMullen C.A., Andrade F.H. (2006). Contractile dysfunction and altered metabolic profile of the aging rat thyroarytenoid muscle. J. Appl. Physiol..

[14] Claflin D.R., Faulkner J.A. (1985). Shortening velocity extrapolated to zero load and unloaded shortening velocity of whole rat skeletal muscle. J. Physiol..

[15] Vaca M., Mora E., Cobeta I. (2015). The Aging Voice: Influence of Respiratory and Laryngeal Changes. Otolaryngol. Head Neck Surg..

[16] Hodge F.S., Colton R.H., Kelley R.T. (2001). Vocal Intensity Characteristics inNormal and Elderly Speakers. J. Voice.

[17] Prakup B. (2012). Acoustic Measures of the Voices of Older Singers and Nonsingers. J. Voice.

[18] Ferrand C.T. (2002). Harmonics-to-Noise Ratio. J. Voice.

[19] Baughman R.P., Loudon R.G. (1986). Sound spectral analysis of voice-transmitted sound. Am. Rev. Respir. Dis..

[20] Titze I.R., Baken R.J., Bozeman K.W., Granqvist S., Henrich N., Herbst C.T., Howard D.M., Hunter E.J., Kaelin D., Kent R.D. (2015). Toward a consensus on symbolic notation of harmonics, resonances, and formants in vocalization. J. Acoust. Soc. Am..

[21] Hillenbrand J., Houde R.A. (1996). Acoustic correlates of breathy vocal quality: Dysphonic voices and continuous speech. J. Speech Hear. Res..

[22] Hillenbrand J., Cleveland R.A., Erickson R.L. (1994). Acoustic correlates of breathy vocal quality. J. Speech Hear. Res..

[23] Delgado-Hernández J., León-Gómez N.M., Izquierdo-Arteaga L.M., Llanos-Fumero Y. (2018). Cepstral analysis of normal and pathological voice in Spanish adults. Smoothed cepstral peak prominence in sustained vowels versus connected speech. Acta Otorrinolaringol. Esp..

[24] Li M., Han K., Narayanan S. (2012). Automatic Speaker Age and Gender Recognition Using Acoustic and Prosodic Level Information Fusion. Comput. Speech Lang..

[25] Spiegl W., Stemmer G., Lasarcyk E., Kolhatkar V., Cassidy A., Potard B., Shum S., Song Y., Xu P., Beyerlein P. Analyzing Features for Automatic Age Estimation on Cross-Sectional Data. Proceedings of the INTERSPEECH 2009, 10th Annual Conference of the International Speech Communication Association.

[26] Stolcke A., Kajarekar S.S., Ferrer L., Shrinberg E. (2007). Speaker Recognition with Session Variability Normalization Based on MLLR Adaptation Transforms. IEEE Trans. Audio Speech Lang. Process..

[27] Berardi M.L., Hunter E.J., Ferguson S.H. (2017). Talker age estimation using machine learning. Proc Meet Acoust.

[28] Zhavoronkov A., Li R., Ma C., Mamoshina P. (2019). Deep biomarkers of aging and longevity: From research to applications. Aging.

[29] Deo R.C. (2015). Machine Learning in Medicine. Circulation.

[30] Costantini G., Todisco M., Perfetti R., Basili R., Casali D. Svm Based Transcription System with Short-Term Memory Oriented to Polyphonic Piano Music. Proceedings of the MELECON 2010—2010 15th IEEE Mediterranean Electrotechnical Conference.

[31] Costantini G., Casali D., Todisco M. An SVM Based Classification Method for EEG Signals. Proceedings of the 14th WSEAS international conference on Circuits.

[32] Van Calster B., Wynants L. (2019). Machine Learning in Medicine. N. Engl. J. Med..

[33] Kockmann M., Burget L., Černocký J. Brno University of Technology System for Interspeech 2010 Paralinguistic Challenge. Proceedings of the INTERSPEECH 2010, 11th Annual Conference of the International Speech Communication Association.

[34] Meinedo H., Trancoso I. Age and Gender Classification Using Fusion of Acoustic and Prosodic Features. Proceedings of the INTERSPEECH 2010, 11th Annual Conference of the International Speech Communication Association.

[35] Přibil J., Přibilová A., Matoušek J. (2017). GMM-based speaker age and gender classification in Czech and Slovak. J. Electr. Eng..

[36] Grzybowska J., Kacprzak S. Speaker Age Classification and Regression Using i-Vectors. Proceedings of the INTERSPEECH 2016, 16th Annual Conference of the International Speech Communication Association.

[37] Sedaghi M. (2009). A Comparative Study of Gender and Age Classification in Speech Signals. Iran. J. Electr. Electron. Eng..

[38] Barkana B.D., Zhou J. (2015). A new pitch-range based feature set for a speaker’s age and gender classification. Appl. Acoust..

[39] Higgins J.P. (2016). Smartphone Applications for Patients’ Health and Fitness. Am. J. Med..

[40] Alameen G. (2007). Review of Audacity computer software. TESL-EJ.

[41] Russell S.J., Norvig P., Davis E. (2010). Artificial Intelligence: A Modern Approach.

[42] Specht D. (1991). A General Regression Neural Network. IEEE Trans. Neural Netw..

[43] Alpaydin E. (2010). Introduction to Machine Learning.

[44] Schuller B., Steidl S., Batliner A., Hirschberg J., Burgoon J.K., Baird A., Elkins A., Zhang Y., Coutinho E., Evanini K. The INTERSPEECH 2016 Computational Paralinguistics Challenge: Deception, Sincerity and Native Language. Proceedings of the INTERSPEECH 2016, 16th Annual Conference of the International Speech Communication Association.

[45] Eyben F., Weninger F., Gross F., Schuller B. (2013). Recent Developments in openSMILE, the Munich Open-Source Multimedia Feature Extractor. Proceedings of the 21st ACM International Conference on Multimedia—MM’13.

[46] Schuller B., Steidl S., Batliner A., Vinciarelli A., Scherer K., Ringeval F., Chetouani M., Weninger F., Eyben F., Marchi E. The INTERSPEECH 2013 Computational Paralinguistics Challenge: Social Signals, Conflict, Emotion, Autism. Proceedings of the INTERSPEECH 2013, 13th Annual Conference of the International Speech Communication Association.

[47] Davis S., Mermelstein P. (1980). Comparison of parametric representations for monosyllabic word recognition in continuously spoken sentences. IEEE Trans. Acoust. Speech Signal Process..

[48] Young S., Kershaw D., Odell J., Ollason D., Valtchev V., Woodland P. (2002). The HTK Book. Camb. Univ. Eng. Dep..

[49] Hermansky H., Morgan N. (1994). RASTA processing of speech. IEEE Trans. Speech Audio Process..

[50] Heman-Ackah Y.D., Michael D.D., Goding G.S. (2002). The relationship between cepstral peak prominence and selected parameters of dysphonia. J. Voice.

[51] Heman-Ackah Y.D., Sataloff R.T., Laureyns G., Lurie D., Michael D.D., Heuer R., Rubin A., Eller R., Chandran S., Abaza M. (2014). Quantifying the cepstral peak prominence, a measure of dysphonia. J. Voice.

[52] Hall M.A., Smith L.A. Practical Feature Subset Selection for Machine Learning. Proceedings of the 21st Australasian Computer Science Conference ACSC’98.

[53] Hall M. (2000). Correlation-Based Feature Selection for Machine Learning. Dep. Comput. Sci..

[54] Fayyad U.M., Irani K.B. (1992). On the handling of continuous-valued attributes in decision tree generation. Mach. Learn..

[55] Platt J. (1999). Fast Training of Support Vector Machines Using Sequential Minimal Optimization. Advances in Kernel Methods: Support Vector Learning.

[56] Frank E., Hall M., Holmes G., Kirkby R., Pfahringer B., Witten I.H., Trigg L., Maimon O., Rokach L. (2009). Weka-A Machine Learning Workbench for Data Mining. Data Mining and Knowledge Discovery Handbook.

[57] DeLong E.R., DeLong D.M., Clarke-Pearson D.L. (1988). Comparing the areas under two or more correlated receiver operating characteristic curves: A nonparametric approach. Biometrics.

[58] Assembly U.G. (1989). Convention on the Rights of the Child. U. N. Treaty Ser..

[59] Livingston G., Huntley J., Sommerlad A., Ames D., Ballard C., Banerjee S., Brayne C., Burns A., Cohen-Mansfield J., Cooper C. (2020). Dementia prevention, intervention, and care: 2020 report of the Lancet Commission. Lancet.

[60] Hegde S., Shetty S., Rai S., Dodderi T. (2019). A Survey on Machine Learning Approaches for Automatic Detection of Voice Disorders. J. Voice.

[61] Zhan A., Mohan S., Tarolli C., Schneider R.B., Adams J.L., Sharma S., Elson M.J., Spear K.L., Glidden A.M., Little M.A. (2018). Using Smartphones and Machine Learning to Quantify Parkinson Disease Severity: The Mobile Parkinson Disease Score. JAMA Neurol..

[62] Arora S., Venkataraman V., Zhan A., Donohue S., Biglan K.M., Dorsey E.R., Little M.A. (2015). Detecting and monitoring the symptoms of Parkinson’s disease using smartphones: A pilot study. Parkinsonism Relat. Disord..

[63] Hakkesteegt M.M., Brocaar M.P., Wieringa M.H., Feenstra L. (2006). Influence of Age and Gender on the Dysphonia Severity Index. Folia Phoniatr. Logop..

[64] Awan S.N. (2006). The aging female voice: Acoustic and respiratory data. Clin. Linguist. Phon..

[65] Ma E.P.-M., Love A.L. (2010). Electroglottographic Evaluation of Age and Gender Effects During Sustained Phonation and Connected Speech. J. Voice.

[66] de Aquino F.S., Ferreira L.P., de Aquino F.S., Ferreira L.P. (2016). Vocal Parameters of Elderly Female Choir Singers. Int. Arch. Otorhinolaryngol..

[67] Deliyski D. (2001). Effects of aging on selected acoustic voice parameters: Preliminary normative data and educational implications. Educ. Gerontol..

[68] Decoster W., Debruyne F. (1997). The ageing voice: Changes in fundamental frequency, waveform stability and spectrum. Acta Otorhinolaryngol. Belg..

[69] Harnsberger J.D., Shrivastav R., Brown W.S., Rothman H., Hollien H. (2008). Speaking rate and fundamental frequency as speech cues to perceived age. J. Voice.

[70] Benjamin B.J. (1981). Frequency variability in the aged voice. J. Gerontol..

[71] Orlikoff R.F. (1990). The Relationship of Age and Cardiovascular Health to Certain Acoustic Characteristics of Male Voices. J. Speech Lang. Hear. Res..

[72] Ramig L.A., Ringel R.L. (1983). Effects of Physiological Aging on Selected Acoustic Characteristics of Voice. J. Speech Lang. Hear. Res..

[73] Zraick R.I., Smith-Olinde L., Shotts L.L. (2012). Adult Normative Data for the KayPENTAX Phonatory Aerodynamic System Model 6600. J. Voice.

[74] Connor N.P., Suzuki T., Sewall G.K., Lee K., Heisey D.M. (2002). Neuromuscular Junction Changes in Aged Rat Thyroarytenoid Muscle. Ann. Otol. Rhinol. Laryngol..

[75] Tiago R.S.L., de Pontes P.A.L., de Brasil O.O.C. (2008). do Quantitative analysis of myelinic fibers in human laryngeal nerves according to age. Braz. J. Otorhinolaryngol..

[76] D’haeseleer E., Depypere H., Claeys S., Baudonck N., Van Lierde K. (2012). The Impact of Hormone Therapy on Vocal Quality in Postmenopausal Women. J. Voice.

[77] Suppa A., Asci F., Saggio G., Marsili L., Casali D., Zarezadeh Z., Ruoppolo G., Berardelli A., Costantini G. (2020). Voice analysis in adductor spasmodic dysphonia: Objective diagnosis and response to botulinum toxin. Parkinsonism Relat. Disord..

[78] Bai X. (2018). Biomarkers of Aging. Adv. Exp. Med. Biol..

[79] Jylhävä J., Pedersen N.L., Hägg S. (2017). Biological Age Predictors. EBioMedicine.

[80] Khan S.S., Singer B.D., Vaughan D.E. (2017). Molecular and physiological manifestations and measurement of aging in humans. Aging Cell.

[81] Hamczyk M.R., Nevado R.M., Barettino A., Fuster V., Andrés V. (2020). Biological Versus Chronological Aging. J. Am. Coll. Cardiol..

[82] Mueller P. (1997). The Aging Voice. Semin Speech Lang..

[83] de Souza L.B.R., Santos M.M.D. (2018). Body mass index and acoustic voice parameters: Is there a relationship?. Braz. J. Otorhinolaryngol..

[84] Suppa A., Marsili L., Giovannelli F., Stasio F.D., Rocchi L., Upadhyay N., Ruoppolo G., Cincotta M., Berardelli A. (2015). Abnormal motor cortex excitability during linguistic tasks in adductor-type spasmodic dysphonia. Eur. J. Neurosci..

