# Supplementary material for: Machine-Learning Analysis of Voice Samples Recorded through Smartphones: The Combined Effect of Ageing and Gender
Source: Sensors (Basel). 2020 Sep 4;20(18):5022. doi: 10.3390/s20185022 (PMC7570582; doi:10.3390/s20185022)
Supplement: Supplementary file 1 [file sensors-20-05022-s001.zip › sensors-889119-supplementary/Table_S1.docx]

**Table S1.** Demographic and anthropometric characteristics of Younger Adults.

| **Subject** | **Gender** | **Age (years)** | **Weight (Kg)** | **Height (cm)** | **BMI** |
| --- | --- | --- | --- | --- | --- |
| 1 | F | 29 | 55 | 164 | 20.4 |
| 2 | F | 28 | 62 | 165 | 22.8 |
| 3 | F | 29 | 54 | 163 | 20.3 |
| 4 | F | 21 | 61 | 165 | 22.4 |
| 5 | F | 25 | 59 | 171 | 20.2 |
| 6 | F | 25 | 52 | 165 | 19.1 |
| 7 | F | 26 | 58 | 167 | 20.8 |
| 8 | F | 25 | 53 | 160 | 20.7 |
| 9 | F | 25 | 68 | 164 | 25.3 |
| 10 | F | 27 | 68 | 152 | 29.4 |
| 11 | F | 25 | 52 | 173 | 17.4 |
| 12 | F | 23 | 63 | 169 | 22.1 |
| 13 | F | 25 | 48 | 164 | 17.8 |
| 14 | F | 27 | 70 | 170 | 24.2 |
| 15 | F | 28 | 43 | 155 | 17.9 |
| 16 | F | 28 | 55 | 165 | 20.2 |
| 17 | F | 29 | 63 | 165 | 23.1 |
| 18 | F | 26 | 60 | 167 | 21.5 |
| 19 | F | 27 | 55 | 170 | 19.0 |
| 20 | F | 21 | 53 | 173 | 17.7 |
| 21 | F | 24 | 52 | 167 | 18.6 |
| 22 | F | 23 | 56 | 163 | 21.1 |
| 23 | F | 29 | 57 | 160 | 22.3 |
| 24 | F | 29 | 50 | 166 | 18.1 |
| 25 | F | 22 | 68 | 171 | 23.3 |
| 26 | F | 19 | 52 | 175 | 17.0 |
| 27 | F | 26 | 52 | 164 | 19.3 |
| 28 | F | 15 | 45 | 154 | 19.0 |
| 29 | F | 25 | 60 | 167 | 21.5 |
| 30 | F | 25 | 58 | 170 | 20.1 |
| 31 | F | 20 | 50 | 165 | 18.4 |
| 32 | F | 25 | 50 | 163 | 18.8 |
| 33 | F | 29 | 53 | 168 | 18.8 |
| 34 | F | 29 | 50 | 169 | 17.5 |
| 35 | F | 29 | 64 | 160 | 25.0 |
| 36 | F | 20 | 54 | 162 | 20.6 |
| 37 | F | 21 | 58 | 173 | 19.4 |
| 38 | F | 20 | 43 | 155 | 17.9 |
| 39 | F | 21 | 56 | 167 | 20.1 |
| 40 | F | 21 | 56 | 175 | 18.3 |
| 41 | F | 22 | 54 | 162 | 20.6 |
| 42 | F | 21 | 69 | 172 | 23.3 |
| 43 | F | 23 | 51 | 165 | 18.7 |
| 44 | F | 28 | 60 | 168 | 21.3 |
| 45 | F | 23 | 55 | 173 | 18.4 |
| 46 | F | 28 | 54 | 166 | 19.6 |
| 47 | F | 29 | 56 | 170 | 19.4 |
| 48 | F | 27 | 56 | 166 | 20.3 |
| 49 | F | 23 | 75 | 187 | 21.4 |
| 50 | F | 23 | 51 | 168 | 18.1 |
| 51 | F | 23 | 59 | 168 | 20.9 |
| 52 | F | 26 | 55 | 170 | 19.0 |
| 53 | F | 24 | 55 | 169 | 19.3 |
| 54 | F | 25 | 57 | 164 | 21.2 |
| 55 | F | 23 | 62 | 163 | 23.3 |
| 56 | F | 25 | 61 | 165 | 22.4 |
| 57 | F | 26 | 44 | 159 | 17.4 |
| 58 | F | 23 | 55 | 167 | 19.7 |
| 59 | F | 25 | 50 | 168 | 17.7 |
| 60 | F | 25 | 68 | 170 | 23.5 |
| 61 | F | 22 | 75 | 175 | 24.5 |
| 62 | F | 24 | 51 | 172 | 17.2 |
| 63 | F | 23 | 52 | 170 | 18.0 |
| 64 | F | 22 | 57 | 163 | 21.5 |
| 65 | F | 28 | 60 | 161 | 23.1 |
| 66 | F | 24 | 53 | 160 | 20.7 |
| 67 | F | 22 | 60 | 160 | 23.4 |
| 68 | F | 28 | 58 | 164 | 21.6 |
| 69 | F | 24 | 43 | 158 | 17.2 |
| 70 | F | 25 | 56 | 178 | 17.7 |
| 71 | F | 24 | 48 | 160 | 18.8 |
| 72 | F | 23 | 48 | 165 | 17.6 |
| 73 | F | 28 | 84 | 163 | 31.6 |
| 74 | M | 29 | 58 | 165 | 21.3 |
| 75 | M | 27 | 59 | 168 | 20.9 |
| 76 | M | 29 | 80 | 186 | 23.1 |
| 77 | M | 28 | 87 | 170 | 30.1 |
| 78 | M | 29 | 73 | 186 | 21.1 |
| 79 | M | 28 | 62 | 162 | 23.6 |
| 80 | M | 27 | 82 | 184 | 24.2 |
| 81 | M | 25 | 63 | 178 | 19.9 |
| 82 | M | 29 | 87 | 175 | 28.4 |
| 83 | M | 25 | 70 | 185 | 20.5 |
| 84 | M | 27 | 80 | 186 | 23.1 |
| 85 | M | 22 | 68 | 165 | 25.0 |
| 86 | M | 28 | 80 | 180 | 24.7 |
| 87 | M | 26 | 60 | 165 | 22.0 |
| 88 | M | 29 | 100 | 178 | 31.6 |
| 89 | M | 30 | 75 | 174 | 24.8 |
| 90 | M | 29 | 80 | 190 | 22.2 |
| 91 | M | 25 | 79 | 180 | 24.4 |
| 92 | M | 25 | 67 | 186 | 19.4 |
| 93 | M | 27 | 70 | 178 | 22.1 |
| 94 | M | 25 | 73 | 185 | 21.3 |
| 95 | M | 30 | 80 | 187 | 22.9 |
| 96 | M | 23 | 73 | 181 | 22.3 |
| 97 | M | 29 | 72 | 172 | 24.3 |
| 98 | M | 28 | 85 | 183 | 25.4 |
| 99 | M | 23 | 65 | 171 | 22.2 |
| 100 | M | 23 | 68 | 173 | 22.7 |
| 101 | M | 27 | 65 | 170 | 22.5 |
| 102 | M | 28 | 78 | 185 | 22.8 |
| 103 | M | 27 | 68 | 171 | 23.3 |
| 104 | M | 29 | 85 | 187 | 24.3 |
| 105 | M | 25 | 76 | 183 | 22.7 |
| 106 | M | 21 | 65 | 180 | 20.1 |
| 107 | M | 23 | 80 | 182 | 24.2 |
| 108 | M | 21 | 79 | 177 | 25.2 |
| 109 | M | 23 | 70 | 182 | 21.1 |
| 110 | M | 23 | 85 | 175 | 27.8 |
| 111 | M | 25 | 80 | 190 | 22.2 |
| 112 | M | 23 | 65 | 182 | 19.6 |
| 113 | M | 21 | 60 | 170 | 20.8 |
| 114 | M | 21 | 56 | 168 | 19.8 |
| 115 | M | 17 | 56 | 167 | 20.1 |
| 116 | M | 21 | 65 | 164 | 24.2 |
| 117 | M | 23 | 78 | 182 | 23.5 |
| 118 | M | 28 | 70 | 177 | 22.3 |
| 119 | M | 29 | 107 | 183 | 32.0 |
| 120 | M | 28 | 78 | 173 | 26.1 |
| 121 | M | 28 | 77 | 174 | 25.4 |
| 122 | M | 28 | 66 | 172 | 22.3 |
| 123 | M | 28 | 99 | 185 | 28.9 |
| 124 | M | 22 | 54 | 168 | 19.1 |
| 125 | M | 26 | 80 | 178 | 25.2 |
| 126 | M | 24 | 67 | 175 | 21.9 |
| 127 | M | 28 | 75 | 174 | 24.8 |
| 128 | M | 29 | 75 | 180 | 23.1 |
| 129 | M | 25 | 80 | 180 | 24.7 |
| 130 | M | 23 | 75 | 187 | 21.4 |
| 131 | M | 26 | 79 | 182 | 23.8 |
| 132 | M | 29 | 72 | 179 | 22.5 |
| 133 | M | 23 | 53 | 170 | 18.3 |
| 134 | M | 19 | 80 | 180 | 24.7 |
| 135 | M | 28 | 70 | 178 | 22.1 |
| 136 | M | 22 | 65 | 173 | 21.7 |
| 137 | M | 25 | 78 | 170 | 27.0 |
| 138 | M | 16 | 62 | 175 | 20.2 |
|  |  |  |  |  |  |
| Av±SD |  | 25.1±3.1 | 64.5±12.4 | 171.4±8.5 | 21.8±3.1 |
