# Supplementary material for: Machine-Learning Analysis of Voice Samples Recorded through Smartphones: The Combined Effect of Ageing and Gender
Source: Sensors (Basel). 2020 Sep 4;20(18):5022. doi: 10.3390/s20185022 (PMC7570582; doi:10.3390/s20185022)
Supplement: Supplementary file 1 [file sensors-20-05022-s001.zip › sensors-889119-supplementary/Table_S2.docx]

**Table S2.** Demographic and anthropometric characteristics of Older Adults.

| **Subject** | **Gender** | **Age (years)** | **Weight (Kg)** | **Height (cm)** | **BMI** |
| --- | --- | --- | --- | --- | --- |
| 1 | F | 54 | 75 | 170 | 26.0 |
| 2 | F | 46 | 55 | 165 | 20.2 |
| 3 | F | 70 | 69 | 165 | 25.3 |
| 4 | F | 65 | 66 | 160 | 25.8 |
| 5 | F | 59 | 60 | 155 | 25.0 |
| 6 | F | 50 | 65 | 150 | 28.9 |
| 7 | F | 61 | 60 | 165 | 22.0 |
| 8 | F | 62 | 60 | 155 | 25.0 |
| 9 | F | 47 | 62 | 168 | 22.0 |
| 10 | F | 56 | 70 | 175 | 22.9 |
| 11 | F | 59 | 79 | 170 | 27.3 |
| 12 | F | 56 | 70 | 160 | 27.3 |
| 13 | F | 46 | 62 | 168 | 22.0 |
| 14 | F | 50 | 62 | 165 | 22.8 |
| 15 | F | 47 | 67 | 177 | 21.4 |
| 16 | F | 70 | 58 | 163 | 21.8 |
| 17 | F | 68 | 65 | 163 | 24.5 |
| 18 | F | 59 | 74 | 156 | 30.4 |
| 19 | F | 77 | 68 | 150 | 30.2 |
| 20 | F | 80 | 65 | 150 | 28.9 |
| 21 | F | 72 | 65 | 153 | 27.8 |
| 22 | F | 77 | 68 | 150 | 30.2 |
| 23 | F | 73 | 85 | 153 | 36.3 |
| 24 | F | 51 | 65 | 150 | 28.9 |
| 25 | F | 50 | 58 | 165 | 21.3 |
| 26 | F | 57 | 110 | 155 | 45.8 |
| 27 | F | 58 | 69 | 168 | 24.4 |
| 28 | F | 46 | 55 | 159 | 21.8 |
| 29 | F | 55 | 57 | 160 | 22.3 |
| 30 | F | 54 | 105 | 167 | 37.6 |
| 31 | F | 51 | 85 | 159 | 33.6 |
| 32 | F | 61 | 52 | 155 | 21.6 |
| 33 | F | 85 | 55 | 154 | 23.2 |
| 34 | F | 70 | 75 | 158 | 30.0 |
| 35 | F | 61 | 70 | 162 | 26.7 |
| 36 | F | 40 | 65 | 175 | 21.2 |
| 37 | F | 44 | 64 | 174 | 21.1 |
| 38 | F | 45 | 75 | 170 | 26.0 |
| 39 | F | 77 | 59 | 156 | 24.2 |
| 40 | F | 57 | 77 | 170 | 26.6 |
| 41 | F | 58 | 57 | 157 | 23.1 |
| 42 | F | 45 | 60 | 166 | 21.8 |
| 43 | F | 77 | 66 | 150 | 29.3 |
| 44 | F | 70 | 61 | 156 | 25.1 |
| 45 | F | 65 | 73 | 165 | 26.8 |
| 46 | F | 61 | 54 | 167 | 19.4 |
| 47 | F | 75 | 53 | 140 | 27.0 |
| 48 | F | 76 | 55 | 157 | 22.3 |
| 49 | F | 77 | 63 | 150 | 28.0 |
| 50 | F | 65 | 66 | 154 | 27.8 |
| 51 | F | 73 | 59 | 158 | 23.6 |
| 52 | F | 71 | 70 | 155 | 29.1 |
| 53 | F | 67 | 50 | 155 | 20.8 |
| 54 | F | 55 | 50 | 158 | 20.0 |
| 55 | F | 57 | 95 | 162 | 36.2 |
| 56 | F | 65 | 65 | 160 | 25.4 |
| 57 | F | 59 | 56 | 160 | 21.9 |
| 58 | F | 51 | 62 | 160 | 24.2 |
| 59 | F | 63 | 58 | 160 | 22.7 |
| 60 | F | 51 | 62 | 157 | 25.2 |
| 61 | F | 51 | 78 | 170 | 27.0 |
| 62 | F | 57 | 80 | 168 | 28.3 |
| 63 | F | 57 | 56 | 164 | 20.8 |
| 64 | F | 42 | 70 | 174 | 23.1 |
| 65 | F | 73 | 72 | 165 | 26.4 |
| 66 | F | 66 | 68 | 160 | 26.6 |
| 67 | F | 59 | 44 | 164 | 16.4 |
| 68 | F | 62 | 57 | 160 | 22.3 |
| 69 | F | 64 | 73 | 162 | 27.8 |
| 70 | F | 52 | 64 | 176 | 20.7 |
| 71 | F | 51 | 52 | 164 | 19.3 |
| 72 | F | 52 | 68 | 168 | 24.1 |
| 73 | F | 60 | 61 | 165 | 22.4 |
| 74 | F | 50 | 60 | 163 | 22.6 |
| 75 | F | 52 | 73 | 160 | 28.5 |
| 76 | F | 47 | 55 | 160 | 21.5 |
| 77 | M | 75 | 70 | 165 | 25.7 |
| 78 | M | 50 | 60 | 170 | 20.8 |
| 79 | M | 48 | 90 | 180 | 27.8 |
| 80 | M | 60 | 83 | 183 | 24.8 |
| 81 | M | 47 | 83 | 173 | 27.7 |
| 82 | M | 78 | 80 | 174 | 26.4 |
| 83 | M | 77 | 85 | 182 | 25.7 |
| 84 | M | 67 | 75 | 168 | 26.6 |
| 85 | M | 51 | 74 | 180 | 22.8 |
| 86 | M | 59 | 69 | 175 | 22.5 |
| 87 | M | 50 | 70 | 175 | 22.9 |
| 88 | M | 85 | 70 | 152 | 30.3 |
| 89 | M | 63 | 75 | 171 | 25.6 |
| 90 | M | 50 | 80 | 175 | 26.1 |
| 91 | M | 50 | 92 | 167 | 33.0 |
| 92 | M | 66 | 63 | 176 | 20.3 |
| 93 | M | 54 | 78 | 177 | 24.9 |
| 94 | M | 81 | 98 | 178 | 30.9 |
| 95 | M | 81 | 70 | 169 | 24.5 |
| 96 | M | 63 | 73 | 174 | 24.1 |
| 97 | M | 60 | 89 | 180 | 27.5 |
| 98 | M | 65 | 80 | 168 | 28.3 |
| 99 | M | 65 | 70 | 160 | 27.3 |
| 100 | M | 66 | 69 | 171 | 23.6 |
| 101 | M | 70 | 63 | 170 | 21.8 |
| 102 | M | 58 | 78 | 175 | 25.5 |
| 103 | M | 58 | 75 | 179 | 23.4 |
| 104 | M | 51 | 65 | 180 | 20.1 |
| 105 | M | 53 | 66 | 170 | 22.8 |
| 106 | M | 46 | 84 | 186 | 24.3 |
| 107 | M | 78 | 79 | 181 | 24.1 |
| 108 | M | 50 | 102 | 186 | 29.5 |
| 109 | M | 52 | 67 | 170 | 23.2 |
| 110 | M | 63 | 88 | 172 | 29.7 |
| 111 | M | 49 | 85 | 185 | 24.8 |
| 112 | M | 50 | 71 | 182 | 21.4 |
| 113 | M | 42 | 79 | 182 | 23.8 |
| 114 | M | 50 | 71 | 175 | 23.2 |
| 115 | M | 41 | 73 | 176 | 23.6 |
| 116 | M | 48 | 85 | 175 | 27.8 |
| 117 | M | 50 | 85 | 170 | 29.4 |
| 118 | M | 49 | 80 | 179 | 25.0 |
| 119 | M | 46 | 67 | 176 | 21.6 |
| 120 | M | 45 | 88 | 177 | 28.1 |
| 121 | M | 47 | 60 | 170 | 20.8 |
| 122 | M | 46 | 76 | 182 | 22.9 |
| 123 | M | 47 | 70 | 185 | 20.5 |
|  |  |  |  |  |  |
| Av±SD |  | 58.9±11.0 | 69.9±11.9 | 166.5±9.8 | 25.2±4.1 |
