# Supplementary material for: Machine-Learning Analysis of Voice Samples Recorded through Smartphones: The Combined Effect of Ageing and Gender
Source: Sensors (Basel). 2020 Sep 4;20(18):5022. doi: 10.3390/s20185022 (PMC7570582; doi:10.3390/s20185022)
Supplement: Supplementary file 1 [file sensors-20-05022-s001.zip › sensors-889119-supplementary/Table_S3.docx]

**Table S3.** Ranking of the first 20 features (functionals applied to low-level descriptors) extracted using OpenSMILE and selected using CAE for the comparison between YA and OA, during the emission of the vowel and the sentence. Each feature is identified by four items: 1) family of low-level descriptor (LLD), 2) LLD, 3) functional used to calculate that specific feature and, 4) the value of relevance calculated through CAE algorithm.

|  | **Vowel** | | | |  | **Sentence** | | | |
| --- | --- | --- | --- | --- | --- | --- | --- | --- | --- |
| **Ranking position** | **Families of LLDs** | **LLDs** | **Functionals** | **Value of Relevance calculated with CAE*** |  | **Families of LLDs** | **LLDs** | **Functionals** | **Value of Relevance calculated with CAE*** |
| 1 | Energy Related LLD | Zero Crossing Rate (de)** | Relative minimum range | 35.042 |  | Energy Related LLD | RMS Energy (de)** | Flatness | 28.021 |
| 2 | Spectral LLD | Spectral Variance (de)** | Range | 34.867 |  | Energy Related LLD | Sum of auditory spectrum (loudness) | 3^rd^ coefficient of the quadratic regression | 27.018 |
| 3 | MFCC | 3^rd^ Mel Coefficient | Root Quadratic Mean | 34.016 |  | Spectral LLD | Spectral Slope (de)** | Flatness | 26.505 |
| 4 | Spectral LLD | Psychoacoustic Sharpness (de)** | Kurtosis | 33.993 |  | Spectral LLD | Spectral Harmonicity (de)** | Flatness | 26.096 |
| 5 | RASTA coefficients | Coefficient of band 25 | Position of minimum | 33.729 |  | MFCC | 1^st^ Mel Coefficient | 3^rd^ coefficient of the quadratic regression | 25.936 |
| 6 | SNR coefficients | SNR | SNR pure values of all the subjects | 33.258 |  | MFCC | 5^th^ Mel Coefficient | 3^rd^ coefficient of the quadratic regression | 24.212 |
| 7 | RASTA coefficients | Coefficient of band 19 | Relative peak range | 33.172 |  | MFCC | 1^st^ Mel Coefficient | 2^nd^ coefficient of the quadratic regression | 22.161 |
| 8 | MFCC | 3^rd^ Mel Coefficient | 3^rd^ coefficient of the quadratic regression | 33.132 |  | Spectral LLD | Spectral Entropy | Mean of peak distances | 21.553 |
| 9 | Spectral LLD | Psychoacoustic Sharpness (de)** | Mean of rising slope | 33.089 |  | Energy Related LLD | RMS Energy | 3^rd^ coefficient of the quadratic regression | 21.521 |
| 10 | Spectral LLD | Spectral Variance (de)** | Relative duration of the LLD is above 75 | 31.845 |  | Spectral LLD | Spectral Entropy | Standard deviation of peak distances | 18.609 |
| 11 | MFCC | 14^th^ Mel Coefficient | Standard deviation of peak distances | 31.562 |  | CPPs coefficients | CPPs | CPPs pure values of all the subjects | 18.065 |
| 12 | Spectral LLD | Spectral Variance (de)** | Mean segment length | 31.320 |  | RASTA coefficients | Coefficient of band 4 | 1^st^ Quartile | 17.885 |
| 13 | Voicing Related LLD | Fundamental Frequency (fo) | Relative duration of the LLD is above 25 | 30.100 |  | RASTA coefficients | Coefficient of band 6 | Relative duration of the LLD is rising | 16.636 |
| 14 | MFCC | 6^th^ Mel Coefficient | Standard deviation of peak distances | 29.610 |  | Spectral LLD | Spectral Entropy (de)** | Standard deviation of peak distances | 16.571 |
| 15 | Spectral LLD | Psychoacoustic Sharpness (de)** | Relative minimum range | 29.219 |  | Spectral LLD | Spectral Roll Off 90 (de)** | Coefficient 2 of the linear prediction | 16.221 |
| 16 | MFCC | 14^th^ Mel Coefficient (de)** | Standard deviation of peak distances | 28.115 |  | Spectral LLD | Spectral Roll Off 90 (de)** | Coefficient 0 of the linear prediction | 14.690 |
| 17 | MFCC | 1^st^ Mel Coefficient | Coefficient 2 of the linear prediction | 27.666 |  | RASTA coefficients | Coefficient of band 0 | Relative minimum range | 13.875 |
| 18 | RASTA coefficients | Coefficient of band 25 (de)** | Relative min range | 26.725 |  | Spectral LLD | Spectral Roll Off 90 (de)** | Coefficient 1 of the linear prediction | 13.607 |
| 19 | MFCC | 6^th^ Mel Coefficient | 3^rd^ Quartile | 24.942 |  | RASTA coefficients | Coefficient of band 15 | Inter-quartile 1-2 | 12.740 |
| 20 | Spectral LLD | Spectral Centroid | Relative duration of the LLD is rising | 24.857 |  | RASTA coefficients | Coefficient of band 18 | Position of minimum | 11.853 |

* relevance refers to value calculated through CAE and expressed in logarithmic scale, for each feature

**the suffix “de” refers to 1st order delta coefficient of the smoothed LLD (delta regression coefficients computed from the feature)

CAE: Correlation Attribute Evaluation; CPPs: Cepstral Prominence Peak smoothed; LLD: Low-Level Descriptor; MFCC: mel frequency cepstral coefficient; OA: Older Adult; SNR: Signal to Noise Ratio; YA: Younger Adult.
